# Prognosis of ovarian cancer subsequent to venous thromboembolism: a nationwide Danish cohort study

**DOI:** 10.1186/1471-2407-6-189

**Published:** 2006-07-17

**Authors:** Mette S Tetsche, Mette Nørgaard, Lars Pedersen, Timothy L Lash, Henrik T Sørensen

**Affiliations:** 1 Department of Clinical Epidemiology, Aarhus University Hospital, 8000 Aarhus, Denmark; 2 Department of Gynecology, Aalborg Hospital, Aarhus University Hospital, 9000 Aalborg, Denmark; 3 Department of Epidemiology, Boston University School of Public Health, Boston, MA 02118, USA

## Abstract

**Background:**

Venous thromboembolism (VTE) is associated with ovarian cancer and may impact the prognosis of ovarian cancer. Our aims were to examine the extent of disease at the time of the diagnosis of ovarian cancer and to estimate the impact of VTE on survival of ovarian cancer.

**Methods:**

We identified 12,835 ovarian cancer patients diagnosed from 1980 to 2003 in the Danish Cancer Registry and obtained information on previous primary VTE diagnosis from the Danish National Hospital Discharge Registry. Ovarian cancer patients with previous VTE related to other cancers, surgery, or pregnancy were excluded. The vital status was determined by linking data to the Civil Registration System.

**Results:**

We identified 50 ovarian cancer patients diagnosed less than 4 months after the VTE and 78 ovarian cancer patients diagnosed more than 4 months after the VTE diagnosis. Advanced stages tended to be more common among patients with VTE. One-year survivals were 44% and 54% among the two VTE groups, compared with 63% among patients without VTE. Adjusted (for age, calendar time, comorbidity, and FIGO-stage) mortality ratios were 1.7 (95% CI = 1.2–2.5) and 1.2 (95% CI = 0.8–1.7), respectively.

**Conclusion:**

Ovarian cancer diagnosed less than four months before VTE is associated with an advanced stage and a poorer prognosis.

## Background

Ovarian cancer has a poor prognosis, and is frequently complicated by venous thromboembolic events (VTE) [1-3]. About 70% of ovarian cancers are diagnosed at an advanced stage [4,5], and advanced stage seems to be associated with higher risk of thromboembolic events [6]. Occasionally, VTE occurs prior to cancer diagnosis and research suggests that VTE may occur as a consequence of an underlying, undiagnosed cancer [7,8].

A recently published, large population-based, study found that the incidence of unprovoked VTE was almost three times higher in the year preceding ovarian cancer diagnosis [9]. However, a paucity of research has investigated the effect of VTE on ovarian cancer prognosis. Sorensen et al [6] reported poorer prognosis among cancer patients following a thrombolic event, however they did not examine the prognosis for individual cancer sites. Moreover, it is not clear how the prognosis of ovarian cancer patients is affected by the timing of the thromboembolic event – i.e. prior to, or simultaneous to, diagnosis with ovarian cancer.

VTE is likely to cause significant morbidity for women subsequently diagnosed with ovarian cancer, and to have serious implications for their clinical care. To examine the impact of VTE on survival of ovarian cancer patients, we present a population-based study estimating the extent of disease at ovarian cancer diagnosis, and the impact of VTE in relation to survival from ovarian cancer.

## Methods

### Setting and design

We conducted a nationwide registry-based cohort study in Denmark with a population of approximately 5.4 million inhabitants. The Danish population has free access to tax-supported medical care. A unique, 10-digit, civil registration number (CPR number) is assigned to all Danish citizens at birth, and this number enables unambiguous linkage between registries. The Civil Registration System is updated daily, with information on vital status (dead or alive), and date of death. The Danish Cancer Registry has recorded all patients in Denmark with malignant neoplasms since 1943 [10]. In this registry, the extent of spread of the ovarian cancer at the time of diagnosis is classified FIGO-stage I to IV [11] or unknown. The Danish National Hospital Discharge Registry [12] includes information about all patients admitted to nonpsychiatric hospitals in Denmark since 1977.

We included patients who had a diagnosis of ovarian cancer (codes 175.0, 175.1, 175.2, 175.3, 375.0, 475.0, or 875.0, in the *International Classification of Diseases, 7^th ^revision*) between January 1^st ^1980 and December 31^st ^2003 [13], recorded in the Danish Cancer Registry. We identified 12,875 patients with an incident diagnosis of ovarian cancer and linked using the CPR number, to the National Hospital Discharge Registry to identify those who had a first time hospitalization with a primary VTE (168 patients) before their cancer diagnosis (VTE coded 45099, 45100, in the *International Classification of Diseases, 8^th ^version*; VTE coded DI260, DI269, DI269A, DI801, DI802, DI802B, DI803, DI803D, DI803E, DI803F, in the *International Classification of Diseases, 10^th ^version*) [14,15]. We excluded patients who had 1) a VTE and previous cancer (11 patients), 2) surgery prior to the incidence of VTE (29 patients), and 3) patients who were pregnant or had given birth less than nine months before, or three months after, the VTE (0 patients). The date of the ovarian cancer diagnosis was the date of the discharge diagnosis registered in the National Hospital Discharge Registry. Patients who developed VTE after the treatment for ovarian cancer were not included. A total of 12,835 ovarian cancer patients were included in the study, of which 128 had a diagnosis of VTE before the ovarian cancer. Information on VTE for these patients was included from 1 January 1977 to 31 December 2003.

Two mutually exclusive cohorts of ovarian cancer patients with VTE were defined according to the interval between the diagnoses of VTE and ovarian cancer. Group 1: patients in whom ovarian cancer was diagnosed less than four months after VTE, and, Group 2: patients in whom ovarian cancer was diagnosed over four months after VTE (range 4 months to 27 years). We chose four months as a cut-off, as research indicates a high incidence of unprovoked cases of VTE less than four months prior to cancer diagnosis [9]. For each of the two cohorts of patients with VTE and ovarian cancer, all other Danish ovarian cancer patients without a discharge diagnosis of VTE served as a control cohort. The follow-up period was at least one year (range 1 to 26 years). Mortality was determined by linkage to the Civil Registration System.

### Statistical analysis

#### FIGO stage associated with VTE

The prevalence of patients with ovarian cancer and VTE who had FIGO stage IV disease was compared with the prevalence of all other ovarian cancer patients who had FIGO stage IV disease by computing the prevalence ratio and its associated 95% confidence intervals (CI) (the prevalence of patients with FIGO stage IV and VTE divided by the prevalence of patients with FIGO stage IV without VTE) adjusted for age.

#### Survival

Patients were followed from the date of the ovarian cancer diagnosis to death or the end of the follow-up period (December 31, 2005), whichever was the sooner. We summarize the survival of ovarian cancer patients using Kaplan-Meier survival curves. The proportion of patients surviving at one-year with and without VTE was calculated along with associated 95% confidence intervals [16].

We used Cox proportional hazards regression to compare the mortality among the cancer patients with and without VTE. We computed the hazard ratio (and associated 95% confidence intervals) as a measure of mortality ratio, separately, for one year and greater than one year post-diagnosis. The diagnostic plot indicated that the mortality hazards were proportional within these time intervals (1 year and subsequent years), but not over the entire time period. We adjusted for age (<50 years, 50–69 years, 70–89 years and 90+ years), year of diagnosis (5-year calendar periods), comorbidity (Charlson Comorbidity Index; 0, 1–2, 3+) [17], and FIGO stage. All estimates were given with 95% confidence intervals, which were calculated by the profile likelihood method. All statistical analyses were performed using SAS (version 9.1.3, SAS Institute, Cary, N.C.). The study was approved by the Aarhus University Registry Board and the Danish Data Protection Agency (record no. 2004-41-4353).

## Results

We identified 128 patients out of 12,835 ovarian cancer patients who were registered with an ovarian cancer diagnosis subsequent to a diagnosis of VTE. Of these 128 patients, 50 were diagnosed with ovarian cancer less than four months after VTE, an additional 78 had ovarian cancer diagnosed more than four months after an episode of VTE. Characteristics of the ovarian cancer patients are shown in table 1.

### FIGO stage associated with VTE

The proportion of patients with ovarian cancer and VTE for whom information on stage of disease was available did not differ much from that of the ovarian cancer patients without VTE for patients diagnosed less than four months (Table 2). The distribution of ovarian cancer stage was slightly shifted toward later stages in the cohorts of women with VTE compared with the cohort of women without VTE. Among ovarian cancer patients diagnosed less than four months after VTE, 34% had stage IV disease, as compared with 31% of ovarian cancer patients without VTE (age-adjusted prevalence ratio = 1.1; 95% CI = 0.8–1.5) (Table 2). Among the patients in whom VTE was diagnosed more than four months before ovarian cancer, 35% had stage IV disease, as compared with 31% of the ovarian cancer patients without VTE (age-adjusted prevalence ratio = 1.1; 95% CI = 1.0–1.5) (Table 2).

### Survival

**Figure 1:**
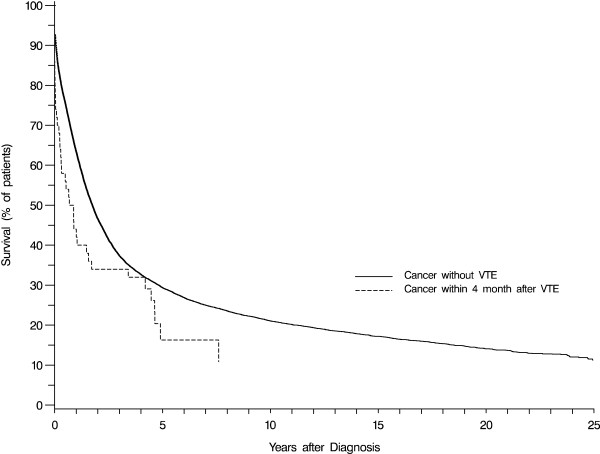
Unadjusted Kaplan-Meier survival curves for patients in whom ovarian cancer was diagnosed less than four months after an episode of VTE versus ovarian cancer patients without VTE.

Figure 1 shows the unadjusted survival curves for patients in whom ovarian cancer was diagnosed less than four months after VTE and all other ovarian cancer patients without VTE. Only 44% (95% CI = 33–60%) of the VTE patients were alive at one year, in contrast to 63% (95% CI = 62–64%) of the comparison cohort. The adjusted mortality ratio was 1.7 (95% CI = 1.2–2.5) for the first year after the cancer diagnosis (Table 3) and 1.0 (95% CI = 0.6–1.8) for the period after the first year.

**Figure 2:**
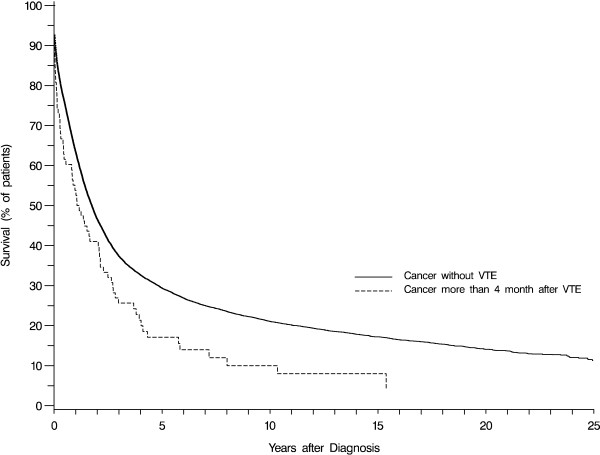
Unadjusted Kaplan-Meier survival curves for patients in whom ovarian cancer was diagnosed more than four months after an episode of VTE versus ovarian cancer patients without VTE.

Patients in whom ovarian cancer was diagnosed more than four months after the episode of VTE also had a poorer prognosis (Figure 2); 54% (95% CI = 44–66%) of them were alive at one year, as compared with 63% (95% CI = 62–64%) of the comparison cohort. The adjusted mortality ratio was 1.2 (95% CI = 0.8–1.7) for the first year of follow-up (Table 3) and 1.1 (95% CI = 0.8–1.6) for the period after the first year.

## Discussion

In this registry-based study of patients with ovarian cancer and VTE, we found that ovarian cancer patients with a concurrent diagnosis of VTE had a poorer prognosis than ovarian cancer patients who did not have VTE. This finding could not be explained by differences between the cohorts in age, calendar-year, comorbidity or FIGO-stage, since the mortality ratio adjusted for these factors did not change substantially from the crude mortality ratio. These findings agree with the limited studies available on the prognosis of patients who have both cancer and VTE reported by Sorensen et al [6].

It seems unlikely that complications of VTE, such as recurrent VTE and pulmonary embolism, can account entirely for the increased mortality rate found in our study. An explanation for our findings could be that the tumours in the VTE patients were more aggressive in some fashion that was not captured by the stage information. About 7% of epithelial ovarian cancers are histopathologically typed as clear cell adenocarcinoma [18]. This type is known to be more aggressive than other types despite being often identified at an early clinical stage [19]. Furthermore, this histopathologic type has also been associated with VTE [20]. However, we did not have access to histopatologic information and could not address this any further.

Our study had other strengths and limitations. We used nationwide population-based registries with no losses to long-term follow-up. Clinicians caring for patients with VTE could have increased their surveillance for cancer in these patients because of the known association with cancer. However, this increased surveillance should have resulted in earlier diagnosis in the patients with VTE compared to ovarian cancer patients without VTE. In addition, patients in whom ovarian cancer was diagnosed more than four months after VTE had a stage distribution shifted towards more advanced stages compared with ovarian cancer patients without VTE. Therefore, surveillance bias is unlikely to be a problem in our study. The ovarian cancer patients were identified in the Danish Cancer Registry, which is known to have a completeness of more than 95% [10]. The diagnosis of VTE can be difficult, however, and the diagnosis from the Danish Hospital Discharge Registry is known to be misclassified in 10 to 20% of the cases [21]. This lack of specificity may lead to an underestimated difference between the patients with VTE and those without it. The FIGO classification was missing in 7% to 13% of the patients. Furthermore, there can be some problems with the validity of the FIGO staging, since not all of the ovarian cancer patients in Denmark are staged and surgically treated by an oncological gynaecologist [22]. These shortcomings allowed for only partial control of differences in tumour aggressiveness, and may account for part of the observed difference in mortality rate.

We lacked information on clinical details such as treatment. If VTE or other diseases associated with VTE contraindicate effective ovarian cancer therapy, then differences in received therapies could explain part of the differences in mortality rates.

In this study we examined all-cause mortality but we did not address cause-specific mortality. However, we find that all-cause mortality is a robust measure, because it is very difficult to distinguish between the contribution of the ovarian cancer and that of the underlying diseases or complications to ovarian cancer.

Another explanation to the increased mortality could be that the coagulation mechanism is implicated in the pathogenesis of cancer. Recent studies suggest that low-molecular-weight-heparin (LMWH) therapy in malignancy may improve cancer survival in general [23-25]. Whereas another study did not find a significantly improved one-year survival in patients with advanced malignancy treated with dalteparin (5,000 IU), a low molecular weight heparin [26]. Our study lacked information about antithrombotic treatment, so we could not examine the impact of the type of antithrombotic treatment on mortality among VTE patients. Our data in combination with the studies of antithrombotics do, however, provide an impetus for further studies of antithrombotic treatment of ovarian cancer patients.

## Conclusion

Our data showed that ovarian cancer diagnosed less than four months before VTE was associated with an advanced stage and the prognosis tends to be poorer than for ovarian cancer patients without VTE. The observed differences in mortality may arise from real differences in the prognosis of ovarian cancer patients with and without VTE, but might also be influenced by the biology of the ovarian cancers associated with VTE. These findings may have implications for the clinical care of patients with ovarian cancer.

## Competing interests

The author(s) declare that they have no competing interests.

## Authors' contributions

MST participated in the design of the study, interpretation of results and has been drafting the manuscript. MN participated in the interpretation of results and helped to draft the manuscript. LP participated in the design of the study, analysis and interpretation of data. TLL participated in the interpretation of results, and revised the manuscript critically. HTS participated in the design of the study, interpretation of results and revised the manuscript critically. All authors read and approved the final manuscript.

## Pre-publication history

The pre-publication history for this paper can be accessed here:



## Figures and Tables

**Table 1:** Characteristics of ovarian cancer patients, N = 12,835.

	**Ovarian cancer without VTE **	**Ovarian cancer less than 4 months after VTE **	**Ovarian cancer 4 months to 27 years after VTE **
	**(N = 12,707)**	**(N = 50)**	**(N = 78)**
**Median age**, years	63	67	71
(25%–75% percentile)	(54–73)	(58–78)	(62–78)
**Age groups**			
<50 years	2,239 (18%)	6 (12%)	9 (12%)
50–69 years	6,328 (50%)	22 (44%)	28 (36%)
70–89 years	4,051 (32%)	22 (44%)	39 (50%)
90+ years	89 (1%)	0 (0%)	2 (3%)
**Year of diagnosis**			
1980–84	2,538 (20%)	10 (20%)	4 (5%)
1985–89	2,711 (21%)	13 (26%)	13 (17%)
1990–94	2,671 (21%)	7 (14%)	20 (26%)
1995–99	2,690 (21%)	7 (14%)	24 (31%)
2000–03	2,097 (17%)	13 (26%)	17 (22%)
**Comorbidity^a^**			
0	11,289 (89%)	47 (94%)	60 (77%)
1–2	1,361 (11%)	3 (6%)	17 (22%)
3+	57 (<1%)	0	1 (1%)

^a ^Charlson Comorbidity Index

**Table 2:** FIGO stage of ovarian cancer, according to the presence or absence of venous thromboembolism.

**Extent of stage**	**Ovarian cancer without VTE **	**Ovarian cancer less than 4 months after VTE **		**Ovarian cancer 4 months to 27 years after VTE **	
	**(n = 12,707)**	**(n = 50)**		**(n = 78)**	
			Prevalence Ratio (95% CI)^a^		Prevalence Ratio (95% CI)^a^

**Patients with data on stage**	11,946 (94%)	47 (94%)		68 (87%)	
Stage I	1,432 (12%)	3 (6%)		9 (13%)	
Stage II	1,600 (13%)	8 (17%)		7 (10%)	
Stage III	5,229 (44%)	20 (43%)		28 (41%)	
Stage IV	3,685 (31%)	16 (34%)	1.1 (0.8–1.5)	24 (35%)	1.1 (0.8–1.5)

Because of rounding, not all column percentages total 100%. Percentages for patients with data on stage are of the entire group of patients. Percentages for patients in the stage categories are of patients with data.

^a^The prevalence ratio is the prevalence of patients with stage IV and VTE divided by the prevalence of patients with stage IV and no VTE, adjusted for age.

**Table 3:** One-year survival and mortality ratios during the first year of follow-up.

	One year survival	Unadjusted mortality ratio	Adjusted mortality ratio ^a^
Ovarian cancer without VTE (control group)	63% (62–64%)		
Ovarian cancer less than 4 months after VTE	44% (33–60%)	1.8 (1.3–2.7)	1.7 (1.2–2.5)
Ovarian cancer 4 months to 27 years after VTE	54% (44–66%)	1.4 (1.0–1.9)	1.2 (0.8–1.7)

^a ^Adjusted for age, calendar-time, comorbidity and FIGO-stage.

Associated 95% confidence interval (CI) is shown in the parenthesis.
